# Molecular modeling and description of a newly characterized activating mutation of the EGFR gene in non-small cell lung cancer

**DOI:** 10.1186/1746-1596-7-146

**Published:** 2012-10-22

**Authors:** Claudia Otto, Agnes Csanadi, Paul Fisch, Martin Werner, Gian Kayser

**Affiliations:** 1 Institute of Pathology, University Medical Center Freiburg, Breisacher Strasse 115a, D-79106, Freiburg, Germany

## Abstract

**Abstract:**

Lung cancer is the leading cause of death among malignant diseases in humans worldwide. In the last decade development of new targeted drugs for the treatment of non-small cell lung cancer proved to be a promising approach to prolong the otherwise very poor prognosis of patients with advanced UICC stages. Epidermal growth factor receptor (EGFR) has been in the focus of this lung cancer science and specific activating mutations are eligible for the treatment with specific tyrosine kinase inhibitors like gefitinib or erlotinib. Beside typical deletions in exon 19 and point mutations in exons 18 and 21 several insertions in exon 19 have been described and attributed activating properties as well. This is the first European and overall the 5^th^ description in English literature of one of these specific insertions. To elucidate its structural changes leading to the activating properties we performed molecular modeling studies. These revealed conformational and electrostatic force field changes in the kinase domain of EGFR. To not miss uncommon mutations thorough and precise characterization of EGFR hotspots, i. e. at least exons 18, 19 and 21, should therefore be conducted to provide best medical care and to offer lung cancer patients appropriate cancer treatment.

**Virtual slides:**

The vistual slides for this article can be found here: http://www.diagnosticpathology.diagnomx.eu/vs/2209889658102062

## Short report

Mutations in the epidermal growth factor receptor (EGFR) are of special diagnostic value in advanced non-small cell lung cancer patients with therapeutic consequences [1-3]. The tyrosine kinase EGFR promotes via KRAS and PI3K mediated pathways tumor cell proliferation, cell survival and escape from apoptosis, while metabolic pathways are shifted towards synthesis of basic cellular elements such as nucleotids, fatty and amino acids [4-7]. Activating mutations in the EGFR gene are located in exons 18 through 21 while more than 90% consist of deletions in exon 19 and L858R substitution in exon 21. These activating mutations are eligible for the treatment with modern tyrosine kinase inhibitors (TKI), e. g. gefitinib or erlotinib [5,8,9]. Just recently, insertions in exon 19 have been described to have an activating character and are responsive to TKI therapy. These shall comprise approximately 1% of EGFR mutations [10]. Described insertions are located at the mutation hotspot of nucleotids 2212 to 2234 thus just prior to the prevalence point of exon 19 deletions [2,10-17].

**Figure 1:**
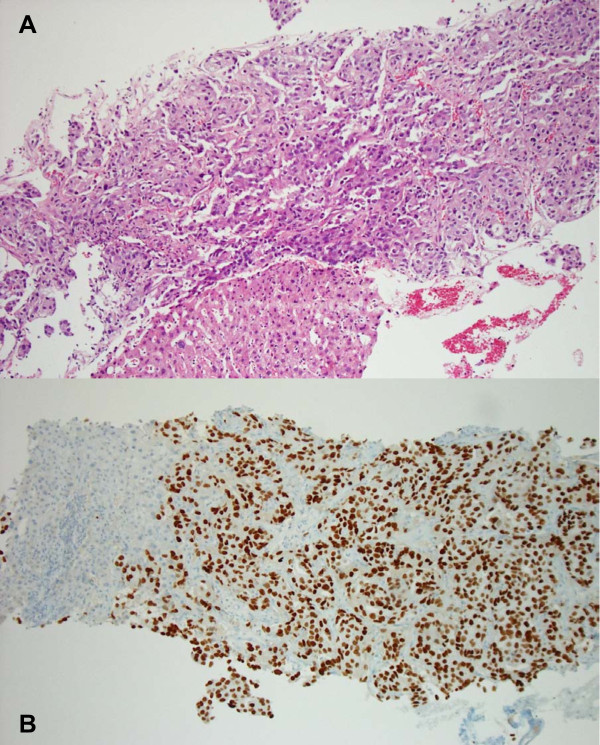
**Histologic morphology of the liver metastasis of the primary lung adenocarcinoma with predominantly solid growth pattern.** (**A** hematoxilin-eosin stain, 10x; **B** immunohistochemical stain for TTF1, 10x).

**Figure 2:**
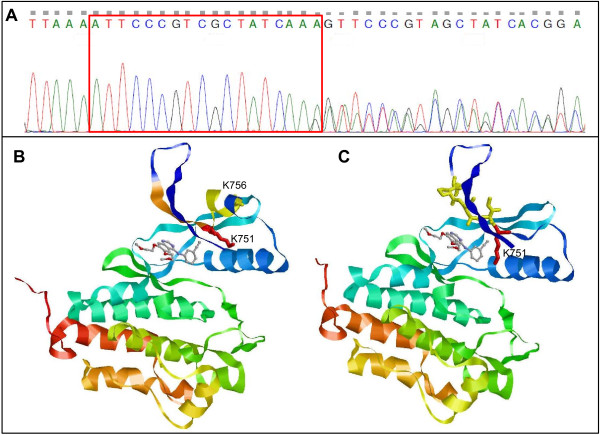
**Molecular characterization of the described insertion in Exon 19 of the *EGFR***-**gene. A**) Sanger sequence of the mutation in exon 19 of the EGFR-gene. Indicated by the red frame are the duplicated 18 nucleotides which do not result in a mutation-typical double curve pattern. As wildtype and mutated DNA strands differ beginning with the end of the insertion/duplication, typical double curve pattern is occurring right behind the mutation. **B**) Molecular model of the mutated protein highlighting the insertion (orange area). Lysin 751 (K751, red) is displaced away from the aromatic ring of gefitinib, thus opening the ATP-binding pocket and changing its electrostatic properties. The 6 aminoacids following the insertion (yellow area) are now in a coiled-coil tertiary structure. **C**) Molecular model of wildtype EGFR in complex with gefitinib. Lysin 751 (K751, red) is in the vicinity of the aromatic ring of gefitinib. The yellow area is highlighting the site of the duplication.

In a 79 year old Kaukasian female patient we were requested to perform EGFR mutation analysis on a liver metastasis of a primary lung adenocarcinoma. The tissue specimen was collected by core biopsy. Histologic examination revealed metastatic formations of a predominantly solid adenocarcinoma. Immunohistochemical nuclear expression of TTF1 proved this to be of primary pulmonary origin (Figure 1). After microdissection of the tumor to enrich neoplastic DNA we performed Sanger sequencing for exons 18, 19 and 21 according to published routine protocol [18]. While exons 18 and 21 were of wildtype sequence this analysis revealed a duplication of 18 nucleotids at position 22147_2235 (c.22147_2235dupl) in exon 19 which leads to the insertion of 6 amino acids (p.K745_E746insIPVAIK) (Figure 2A). Uncommon for EGFR mutations in non-small cell lung cancer the female patient presented here was of older age. 

Since this is the 5^th^ description of this specific duplication in exon 19 of the EGFR-gene in English literature and to our knowledge the first in Europe molecular modeling studies were performed using the SWISS-Prot server [19,20] to investigate the structural changes of this mutation. Crystal structures of wildtype EGFR kinase domain without ligand (protein data bank entry 2gs2) and in complex with gefitinib (protein databank entry 1m17) served as templates for the homology modeling calculations. Visualization confirmed conformational changes in the ATP-binding pocket due to this insertion (Figure 2B and C). Most prominent is the displacement of the side chain residue of Lysin at position 751 (K751). In the wildtype structure this polar side chain is in the vicinity of the hydrophobic aromatic ring of gefitinib. Our modeling results indicate that due to the mutation the hydrophilic residue of Lysin751 opens the binding pocket. This may result in an alteration of electrostatic forces which could lead to a facilitated binding of gefitinib (Figure 2B and C).

Insertions at this site of the EGFR gene result in a change in the ATP-binding pocket allowing a facilitated activation of effector proteins like RAS or PI3Kinase to promote tumor cell proliferation and survival [10,21]. The conformational changes observed here could also explain these published capabilities of exon 19 insertions.

Since exon 19 and especially this subregion of the exon displays a hotspot for genetic mutations of the EGFR gene specific DNA structures at this location on chromosome 7 must prevail. Amongst those small loops due to an adenosine and thymidine rich DNA segment at this location can be suspected to enable cross links in between the same or the complementary DNA-strand [22]. These cross links may then not only result in deletions, which are the most common mutations at this site of exon 19, but also in a duplication, as described here of 18 nucleotides.

Several methods for the evaluation of EGFR-status in non-small cell lung cancer have been described [23-30]. Nevertheless contemporarily new mutations are being detected and their biological impact is in the focus of scientific research. Thus, complete sequencing of the mutation hotspots, i. e at least EGFR exons 18, 19 and 21, leads not only to new insights in tumor biology but also has great therapeutical impact for the patient. Only complete sequenicing will detect new or uncommon mutations which may also respond to TKI therapy.

Concluding, insertions in exon 19 are likely to respond to TKI therapy. Since not only activating mutations of the EGFR gene exist, but secondary resistance to TKI-therapy can be a result of additional mutations thorough analysis and exact characterization of EGFR mutations by sequence analysis in non-small cell lung cancer should be performed.

## Ethics

Data publication is in concordance with the decision of the ethics committee of the University Medical Center Freiburg (EK 10/12).

## Competing interests

The authors state that no conflict of interest exists.

## Authors’ contributions

Clinical assessment and mutation analysis CO and GK; scientific workup and molecular modeling CO, GK; manuscript preparation CO, GK; proofreading: CO, AC, PF, GK, MW. All authors read and approved the final manuscript.

## References

[B1] Toyooka S Kiura K Mitsudomi T EGFR mutation and response of lung cancer to gefitinib N Eng J Med 2005 352 20 2136 author reply 2136 10.1056/NEJM200505193522019 15901872

[B2] Uruga H Kishi K Fujii T Beika Y Enomoto T Takaya H Miyamoto A Morokawa N Kurosaki A Yoshimura K Efficacy of gefitinib for elderly patients with advanced non-small cell lung cancer harboring epidermal growth factor receptor gene mutations: a retrospective analysis Intern Med 2010 49 2 103 107 10.2169/internalmedicine.49.2531 20075572

[B3] Maemondo M Inoue A Kobayashi K Sugawara S Oizumi S Isobe H Gemma A Harada M Yoshizawa H Kinoshita I Gefitinib or chemotherapy for non-small-cell lung cancer with mutated EGFR N Eng J Med 2010 362 25 2380 2388 10.1056/NEJMoa0909530 20573926

[B4] Arteaga CL Epidermal growth factor receptor dependence in human tumors: more than just expression? Oncologist 2002 7 Suppl 4 31 39 12202786 10.1634/theoncologist.7-suppl_4-31

[B5] Gazdar AF Activating and resistance mutations of EGFR in non-small-cell lung cancer: role in clinical response to EGFR tyrosine kinase inhibitors Oncogene 2009 28 Suppl 1 S24 S31 19680293 10.1038/onc.2009.198 PMC2849651

[B6] Kayser G Sienel W Kubitz B Mattern D Stickeler E Passlick B Werner M Zur Hausen A Poor outcome in primary non-small cell lung cancers is predicted by transketolase TKTL1 expression Pathology 2011 43 7 719 724 10.1097/PAT.0b013e32834c352b 22027741

[B7] Shigematsu H Lin L Takahashi T Nomura M Suzuki M Wistuba II Fong KM Lee H Toyooka S Shimizu N Clinical and biological features associated with epidermal growth factor receptor gene mutations in lung cancers J Natl Cancer Inst 2005 97 5 339 346 10.1093/jnci/dji055 15741570

[B8] Shigematsu H Gazdar AF Mutations of EGFR in lung cancers and their implications for targeted therapy Discov Med 2004 4 24 444 447 20704946

[B9] Shigematsu H Gazdar AF Somatic mutations of epidermal growth factor receptor signaling pathway in lung cancers Int J Cancer & Journal International Du Cancer 2006 118 2 257 262 10.1002/ijc.21496 16231326

[B10] He M Capelletti M Nafa K Yun CH Arcila ME Miller VA Ginsberg MS Zhao B Kris MG Eck MJ EGFR exon 19 insertions: a new family of sensitizing EGFR mutations in lung adenocarcinoma Clinical Can Res 2012 18 6 1790 1797 10.1158/1078-0432.CCR-11-2361 PMC3306520 22190593

[B11] De Pas T Toffalorio F Manzotti M Fumagalli C Spitaleri G Catania C Delmonte A Giovannini M Spaggiari L de Braud F Activity of epidermal growth factor receptor-tyrosine kinase inhibitors in patients with non-small cell lung cancer harboring rare epidermal growth factor receptor mutations Journal of Thoracic Oncology 2011 6 11 1895 1901 10.1097/JTO.0b013e318227e8c6 21841502

[B12] Ilie MI Hofman V Bonnetaud C Havet K Lespinet-Fabre V Coelle C Gavric-Tanga V Venissac N Mouroux J Hofman P Usefulness of tissue microarrays for assessment of protein expression, gene copy number and mutational status of EGFR in lung adenocarcinoma Virchows Archiv 2010 457 4 483 495 10.1007/s00428-010-0963-z 20803030

[B13] Job B Bernheim A Beau-Faller M Camilleri-Broet S Girard P Hofman P Mazieres J Toujani S Lacroix L Laffaire J Genomic aberrations in lung adenocarcinoma in never smokers PLoS One 2010 5 12 e15145 10.1371/journal.pone.0015145 21151896 PMC2997777

[B14] Kosaka T Yatabe Y Endoh H Kuwano H Takahashi T Mitsudomi T Mutations of the epidermal growth factor receptor gene in lung cancer: biological and clinical implications Cancer Res 2004 64 24 8919 8923 10.1158/0008-5472.CAN-04-2818 15604253

[B15] Mitsudomi T Kosaka T Endoh H Horio Y Hida T Mori S Hatooka S Shinoda M Takahashi T Yatabe Y Mutations of the epidermal growth factor receptor gene predict prolonged survival after gefitinib treatment in patients with non-small-cell lung cancer with postoperative recurrence J Clin Oncol 2005 23 11 2513 2520 10.1200/JCO.2005.00.992 15738541

[B16] Okami J Taniguchi K Higashiyama M Maeda J Oda K Orita N Koizumi K Kodama K Kato K Prognostic factors for gefitinib-treated postoperative recurrence in non-small cell lung cancer Oncology 2007 72 3–4 234 242 18176089 10.1159/000112947

[B17] Yoshida Y Shibata T Kokubu A Tsuta K Matsuno Y Kanai Y Asamura H Tsuchiya R Hirohashi S Mutations of the epidermal growth factor receptor gene in atypical adenomatous hyperplasia and bronchioloalveolar carcinoma of the lung Lung Cancer 2005 50 1 1 8 10.1016/j.lungcan.2005.04.012 15950315

[B18] Lynch TJ Bell DW Sordella R Gurubhagavatula S Okimoto RA Brannigan BW Harris PL Haserlat SM Supko JG Haluska FG Activating mutations in the epidermal growth factor receptor underlying responsiveness of non-small-cell lung cancer to gefitinib N Eng J Med 2004 350 21 2129 2139 10.1056/NEJMoa040938 15118073

[B19] Arnold K Bordoli L Kopp J Schwede T The SWISS-MODEL workspace: a web-based environment for protein structure homology modelling Bioinformatics 2006 22 2 195 201 10.1093/bioinformatics/bti770 16301204

[B20] Kiefer F Arnold K Kunzli M Bordoli L Schwede T The SWISS-MODEL Repository and associated resources Nucleic Acids Res 2009 37 Database issue D387 D392 18931379 10.1093/nar/gkn750 PMC2686475

[B21] Kumar A Petri ET Halmos B Boggon TJ Structure and clinical relevance of the epidermal growth factor receptor in human cancer J Clin Oncol 2008 26 10 1742 1751 10.1200/JCO.2007.12.1178 18375904 PMC3799959

[B22] Yang W Structure and mechanism for DNA lesion recognition Cell Res 2008 18 1 184 197 10.1038/cr.2007.116 18157156

[B23] Casorzo L Corigliano M Ferrero P Venesio T Risio M Evaluation of 7q31 region improves the accuracy of EGFR FISH assay in non small cell lung cancer Diagn Pathol 2009 4 36 10.1186/1746-1596-4-36 19889201 PMC2781797

[B24] Shen S Qin D Pyrosequencing data analysis software: a useful tool for EGFR, KRAS, and BRAF mutation analysis Diagn Pathol 2012 7 56 10.1186/1746-1596-7-56 22640803 PMC3433338

[B25] Fassina A Gazziero A Zardo D Corradin M Aldighieri E Rossi GP Detection of EGFR and KRAS mutations on trans-thoracic needle aspiration of lung nodules by high resolution melting analysis J Clin Pathol 2009 62 12 1096 1102 10.1136/jcp.2009.067587 19640859

[B26] Yamamoto H Toyooka S Mitsudomi T Impact of EGFR mutation analysis in non-small cell lung cancer Lung Cancer 2009 63 3 315 321 10.1016/j.lungcan.2008.06.021 18760859

[B27] Do H Krypuy M Mitchell PL Fox SB Dobrovic A High resolution melting analysis for rapid and sensitive EGFR and KRAS mutation detection in formalin fixed paraffin embedded biopsies BMC Cancer 2008 8 142 10.1186/1471-2407-8-142 18495026 PMC2408599

[B28] Raz DJ Jablons DM EGFR expression and mutational analysis as a predictive test J Clin Oncol 2007 25 15 2144 2145 17513827 10.1200/JCO.2006.10.1733

[B29] Daniele L Macri L Schena M Dongiovanni D Bonello L Armando E Ciuffreda L Bertetto O Bussolati G Sapino A Predicting gefitinib responsiveness in lung cancer by fluorescence in situ hybridization/chromogenic in situ hybridization analysis of EGFR and HER2 in biopsy and cytology specimens Mol Cancer Ther 2007 6 4 1223 1229 10.1158/1535-7163.MCT-06-0719 17406029

[B30] Soung YH Lee JW Kim SY Seo SH Park WS Nam SW Song SY Han JH Park CK Lee JY Mutational analysis of EGFR and K-RAS genes in lung adenocarcinomas Virchows Archiv 2005 446 5 483 488 10.1007/s00428-005-1254-y 15815931

